# Dermatological guidelines for monitoring methotrexate treatment reduce drug-survival compared to rheumatological guidelines

**DOI:** 10.1371/journal.pone.0194401

**Published:** 2018-03-23

**Authors:** Felien T. M. Busger op Vollenbroek, Carine J. M. Doggen, René W. A. Janssens, Hein J. Bernelot Moens

**Affiliations:** 1 Department of Rheumatology, Ziekenhuisgroep Twente, Almelo, The Netherlands; 2 Health Technology and Services Research, University of Twente, Enschede, The Netherlands; VU University Medical Center, NETHERLANDS

## Abstract

**Background:**

Methotrexate (MTX) is widely used as disease modifying treatment for psoriasis and psoriatic arthritis (PsA). Rheumatological and dermatological guidelines to prevent MTX-induced adverse events diverge in the number and frequency of blood tests. These differences are not based on evidence indicating a higher risk for patients with psoriasis compared to PsA or rheumatic arthritis (RA). This raises the question if multiple testing increases safety, or results in false positive signals potentially leading to early withdrawal of an effective treatment.

**Objective:**

Compare the effects of MTX monitoring strategies by rheumatologists and dermatologists regarding drug survival, reasons for withdrawal and safety.

**Methods:**

Retrospective follow-up of all patients diagnosed with psoriasis by dermatologists or PsA by rheumatologists. Included were consecutive patients who started methotrexate (MTX) between 2006 and 2012 and had a scheduled follow-up by dermatologist or rheumatologist. Exclusions were: drug not started after the first prescription or incomplete availability of lab data. Data were extracted from electronic records: start and stop dates and dosing of MTX; treatment with folic acid and dose; reasons for withdrawal of MTX; numbers of blood sampling and types of laboratory tests performed for MTX safety; number of abnormal tests; occurrence of any serious adverse event (SAE).

**Results:**

190 Psoriasis and 196 PsA patients starting methotrexate (MTX) were included. Age and sex were comparable. PsA patients used higher initial and maximum doses of MTX and folic acid, but psoriasis patients had a higher frequency of abnormal laboratory results (0.14 vs 0.03 per treatment month, p<0.001), resulting in a striking difference in withdrawal of MTX for abnormal liver enzymes (15.8% vs 3.6%, p<0.001). In PsA patients MTX was withdrawn less frequently for ineffectiveness (15.8 vs 24.2%, p<0.05) leading to longer drug survival in the first course of treatment (37.4±30 vs 18.8±19.1 months). Repeated courses of MTX were more often prescribed by rheumatologists than by dermatologists. There were no significant differences in the occurrence of SAE (psoriasis 0.0041 vs PsA 0.0038 per treatment month) or death (1.6% vs 2.0%) between these groups. Hospital admissions related to infection were recorded in 6 (3.1%) PsA vs 4 (2.1%) psoriasis patients.

**Conclusion:**

Strict monitoring by dermatologists resulted in more abnormal findings, which reduced drug survival of MTX. The limited monitoring strategy by rheumatologists was not associated with more SAEs. Further research in other populations is needed to confirm whether reduced intensity of monitoring can optimize the use of MTX with sufficient long-term safety.

## Introduction

Methotrexate (MTX) is widely used as disease modifying treatment for psoriasis and psoriatic arthritis (PsA). Its effectiveness has been demonstrated in randomised trials which prompted adoption of MTX in guidelines.[1–3] he use of MTX is limited by adverse events such as infections, bone marrow suppression, gastrointestinal toxicity and liver toxicity, including fibrosis and cirrhosis.[2,4] Discontinuation of treatment due to adverse events was reported in one third of patients with PsA and psoriasis, mostly due to gastrointestinal side effects.[5,6] Toxic effects on liver and bone marrow are potentially dangerous, and therefore guidelines recommend monitoring of blood counts and liver enzymes.[3,7–10]

Such guidelines were originally based on the use of high dose MTX in cancer treatment. The first guidelines to monitor low dose weekly administration of MTX in psoriasis and rheumatoid arthritis (RA) included blood tests every 4 weeks and liver biopsies.[11,12] Formal evaluation of MTX guidelines has not been reported. Rheumatological guidelines were gradually simplified based on the observation that monitoring resulted in a low frequency of abnormal results, and that serious damage to the liver undisputedly caused by MTX occurs in less than 1% or even less than 0.1% of the patients. [4,13–16] The burden for patients might not outweigh the potential damage. Liver biopsies were therefore limited to high risk patients in rheumatological guidelines, which was followed with some hesitation by dermatologists. Liver biopsies were replaced by blood tests for liver fibrosis, serum amino-terminal propeptide of type III procollagen (PIIIPN), based on results of small studies.[12] The effectiveness of folic acid to prevent liver and bone marrow toxicity was demonstrated in several randomized controlled trials (RCTs), which prompted the inclusion of various doses of folic acid in guidelines for MTX treatment.[17,18]

Current dermatological and rheumatological guidelines to prevent toxicity of MTX diverge in the frequency and type of blood tests. These differences are not based on evidence indicating a higher risk for patients with psoriasis compared to PsA or Rheumatoid arthritis (RA).[13,19,20] This raised a key question: what is the optimal method for screening and monitoring for methotrexate toxicity?[11] Does the gradual reduction of tests by rheumatologists result in unobserved or unexpected adverse events? Or does strict monitoring by dermatologists result in false signals and early withdrawal of a potentially helpful drug? Since a prospective trial to compare the outcome of monitoring strategies is hard to realise, we have analysed a retrospective cohort of patients treated with methotrexate for psoriasis by dermatologists and with PsA treated by rheumatologists using their respective guidelines.

The first aim of this study was to establish whether different monitoring strategies lead to differences in drug survival and reasons for withdrawal of methotrexate. The second aim was to assess the safety of both monitoring protocols.

## Patients and methods

In this retrospective follow-up study the population consists of all patients with a diagnosis of psoriasis by a dermatologist or PsA by a rheumatologist in a large teaching hospital in the Netherlands. The respective monitoring guidelines diverge particularly in the frequency of testing and number of liver enzymes to be tested (Table 1). In the hospital data system we identified all patients with a diagnosis of psoriasis (N = 3959) or PsA (N = 471) seen from 2006 to 2014. We included patients who were advised to start methotrexate (MTX) between January 1, 2006 and December 31, 2012 and had scheduled at least three follow-up visits to a dermatologist or rheumatologist. By review of the electronic records we applied the following exclusion criteria: patient decided not to start the drug after the first prescription; diagnosis of psoriasis or PsA rejected; follow-up by other specialist not using the specific guideline; incomplete availability of lab data; lost to follow-up within 3 months for reasons not related to drug or disease.

**Table 1:** Methotrexate monitoring guidelines used by dermatologists for psoriasis and by rheumatologists for psoriatic arthritis.

	Dermatology guideline [9]	Rheumatology guideline [10]
**Starting dose MTX**	5–10 mg-week	10–15 mg/week
**Highest dose MTX**	Oral 22,5 or SC 30 mg/wk	30 mg/week
**Folic acid**	5–10 mg/week	5–10mg/week
**Initial monitoring**	every two weeks	monthly
**Monitoring long-term**	every 2–3 months	every 3–4 months
**Lab tests**	ALAT, AF, γ-GT, full blood count, kreatinine	ALAT, Hb, leukocytes, thrombocytes, kreatinine
**Advice when abnormal lab results**	Reduce or withdraw when: leukocytes<3,0 or thrombocytes<100;Liver enzymes >2 times upper limit	Reduce or stop when: leukocytes<3,0 or thrombocytes<100 or ALAT>3ULN; withdraw when 2 times: ALAT>3 times upper limit
**Liver biopsy/PIIIPN**	Biopsy on clinical indication; PIIIPN 1x/3months	Biopsy on clinical indication; No PIIIPN

We extracted the following data from the records: patient characteristics; start and stop dates and dosing of MTX; treatment with folic acid and dose; reasons for withdrawal of MTX; numbers of blood sampling and laboratory tests performed for MTX safety; number of abnormal tests; occurrence of any serious adverse event. Data were anonymised for further analysis.

The start date of MTX was the date a first prescription was marked in the records. A stop date was marked when MTX was withdrawn for at least three months, and the reason for withdrawal was recorded. Restart of MTX after three months was seen as a new course. Observations were censored at the date of the last registered hospital visit prior to December 31, 2014 (date of last observation). Drug survival was calculated as the number of days that patients continued treatment with MTX. The reasons for MTX discontinuation were retrieved from the records and categorized as: ineffectiveness, disease remission; abnormality in laboratory results; toxicity related to methotrexate; patient preference; comorbid conditions e.g. treatment for a malignancy or cerebral infarction. Serious adverse event (SAE) included: death, hospitalization, significant disability or damage. All SAEs occurring while a patient was using MTX were retrieved from the hospital records. Laboratory results were recorded as abnormal when they were outside the normal reference ranges, using specified levels for women and men. For leucocytes: ≤4.0*10^9^/l and thrombocytes: ≤150*10^9^/l.

Finally, to assess the number and duration of treatments with biologicals, hospital records of all patients were checked for the use of any biological during or after treatment with MTX between 2006 and May 31, 2015.

The study is deemed to be non-intrusive and therefore does not fall under the Dutch law governing scientific research with humans. Informed consent from the patients was not required, since this was a retrospective follow-up of data registered in the hospital data system, obtained in routine care.

### Statistical analyses

Descriptive data are presented as number and percentages or mean values with standard deviations (SD) and minimum and maximum values. Group differences were tested using chi-square test or by Fisher’s exact test for categorical and t-test for continuous variables which were normally distributed. 95% Confidence intervals (CI) were calculated. Kaplan-Meier plots were used to assess drug survival and log-rank tests were used to assess differences between groups. Hazard ratios (HR) were calculated to indicate risks with 95% CI. Analyses were performed with SPSS version 23 (Chicago, Ill, US). The raw dataset is online available using the following persistent identifier: https://doi.org/10.17026/dans-xvx-ys47

## Results

### Baseline characteristics

**Fig 1:**
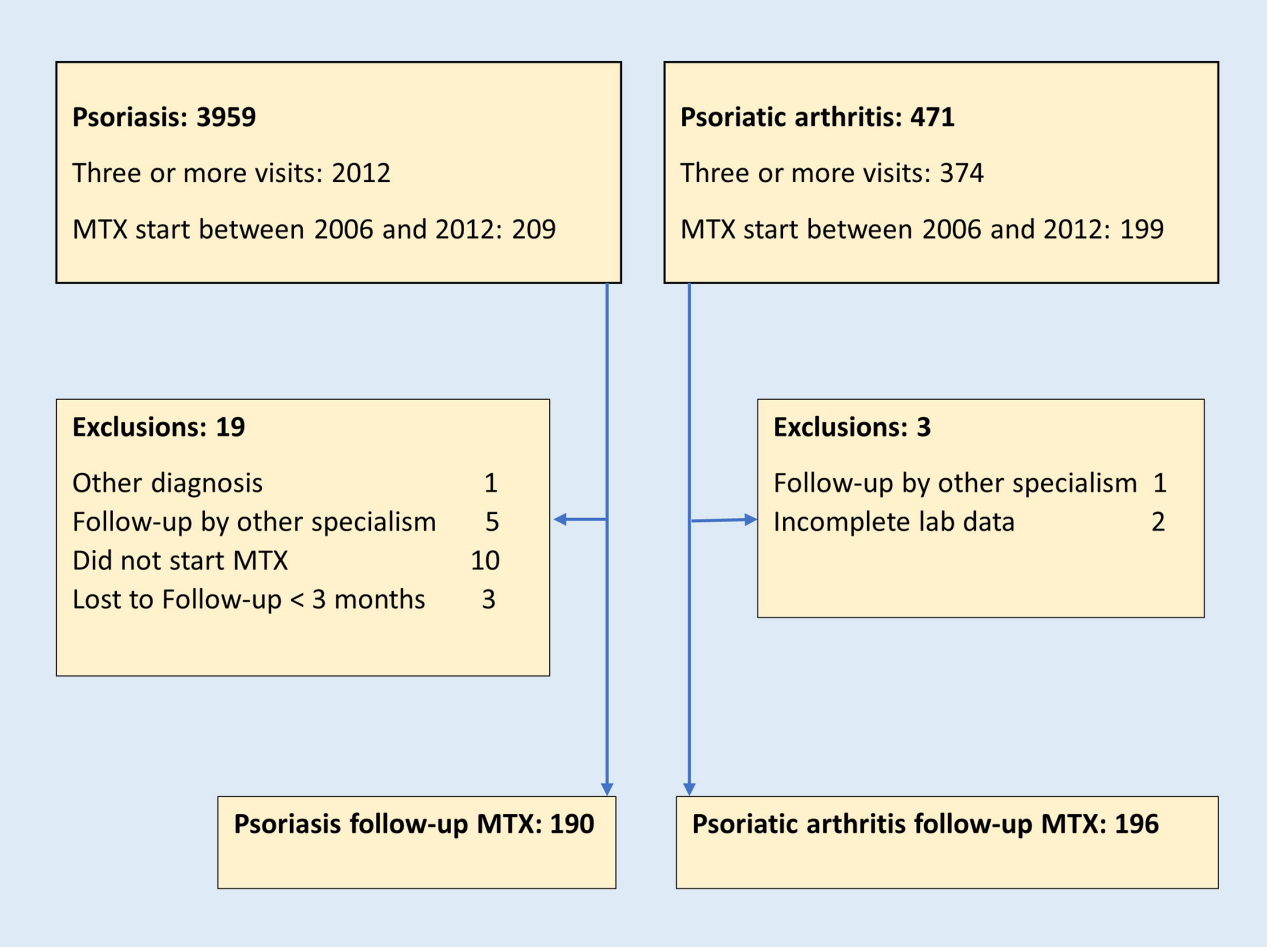
Selection, inclusion and exclusion of study population from the hospital database: Patients seen at least once between 2006 and 2015.

Selection and exclusions of patients are presented in Fig 1. Since many psoriasis patients respond to local or UVB photo-therapy, it is not unexpected that fewer psoriasis patients with three or more visits to the outpatient clinic (209/2012: 9%) were started on methotrexate compared to PsA patients (199/374: 53%). Baseline characteristics of the study population starting MTX between 2006 and 2012 are presented in Table 2. Among 190 psoriasis patients treated by a dermatologist, 8 (4.2%) had a prior course of treatment with MTX. This contrasts with earlier MTX treatment in 39 (19.9%) of PsA patients. The tendency of rheumatologists to prescribe repeated courses of MTX is also illustrated by a higher number of subsequent courses (35.7% vs 6.8% by dermatologists). Psoriasis patients were not treated with a combination of disease modifying drugs. Seven of 190 patients with psoriasis developed PsA during follow-up and were referred after 5.7 (SD 3.6) months of MTX treatment by the dermatologist to the rheumatologist. In these patients no SAEs occurred, and follow-up by a rheumatologist was excluded from further analysis. Co-medication with hydroxychloroquine, sulphasalazine or prednisolone was used by 24 (12%) of the PsA patients, and in one patient methotrexate was combined with adalimumab.

**Table 2:** Characteristics of patients with psoriasis or psoriatic arthritis.

	Psoriasis	Psoriatic arthritis
	Treatment started by dermatologist	Treatment started by rheumatologist
	N = 190	N = 196
Men, N (%)	86 (45.3)	95 (48.5)
Age (y)	52.3 (16.2), 17–94	51.8 (13.8), 16–79
Prior treatment with MTX, N (%)	8 (4.2)	39 (19.9)**
MTX starting dose (mg/week)	12.2 (3.7), 2.5–15	15.2 (3.0), 5–30**
MTX maximal dose (mg/week)	15.6 (4.0), 5–25	21.5 (6.0), 5–40**
Folic acid starting dose (mg/week)	4.9 (0.7), 2.5–10	8.2 (5.4), 5–30**
Folic acid maximal dose (mg/week)	5.6 (2.9), 2.5–30	16.5 (10.4), 5–30**
Duration of first observed MTX course (months)	18.8 (19.1)	37.4 (30.0) **
Patients with 2 or more MTX courses, N (%)	12 (6.3)	25 (12.7) **

mean (SD), min-max unless otherwise indicated, * P value < = 0.05;

** P-value <0.001

The starting MTX dose was 3.0 (95% CI 2.6–4.3) mg per week higher in patients with PsA compared to patients with psoriasis. Similarly, the starting folic acid dose was 3.3 (95% CI 2.4–3.8) mg per week higher in patients with PsA. The first course of MTX in this analysis was administered subcutaneously in 3 psoriasis and in 12 PsA patients. Later courses were administered subcutaneously in 3 and 32 patients respectively, rheumatologists using this route in more patients (22% vs 3%) than dermatologists.

### Drug survival

The first MTX treatment in the 190 psoriasis patients was stopped before the date of last observation in 161 (84.7%) compared to 120 out of 196 (61.2%) of PsA patients. Duration of the first treatment course was much shorter in psoriasis patients (average 18.8 months) than in PsA patients (37.4 months). During the first treatment course the average number of laboratory visits per treatment month was 0.58 in psoriasis patients versus 0.51 in PsA patients (Table 3). Dermatologists ordered more tests per visit in accordance with their protocol. The average number of abnormal laboratory results per treatment month was significantly higher in psoriasis (0.14) than in PsA patients (0.03) (p<0.001). The details for specific tests in Table 3 demonstrate that, whereas the number of patients with any abnormal result for ALT was equal in both groups, monitoring of four different liver enzymes by dermatologists led to a higher number of abnormal results for γ-GT and AST. The majority of the elevations of ALT were mild. In 20 (10.2%) of PsA and 28 (14.4%) of psoriasis patients the elevation of ALT exceeded two times the upper level of normal.

**Table 3:** Laboratory monitoring during first treatment course with MTX.

	Psoriasis	Psoriatic arthritis
	treatment by dermatologist	treatment by rheumatologist
	N = 190	N = 196
Number of laboratory visits	1353	2609
Laboratory visits per treatment month	0.58 (0.74)	0.51 (0.34)
Number of abnormal laboratory results	227	121
Abnormal laboratory results per treatment month	0.14 (0.26)	0.03 (0.07) **
Abnormal laboratory results per laboratory visit ^a^	0.26 (0.39)	0.06 (0.11)**
Any abnormal result N (%)	116 (61.1)	98 (50.0)*
alanine aminotransferase (ALT), N (%)	82 (43.2)	85 (43.4)
aspartate aminotransferase (AST), N (%)	48 (25.3)	4 (2.0)**
γ-glutamyl transpeptidase (γ-GT), N (%)	63 (33.2)	4 (2.0)**
Alkaline phosphatase (AP), N (%)	7 (3.7)	3 (1.5)
Leucocytes, N (%)	7 (3.7)	13 (6.6)
Thrombocytes, N (%)	2 (1.1)	7 (3.6)

Laboratory visits and abnormal laboratory results are presented as mean (SD)

* P value < = 0.05

** P-value <0.001

^a^ Three patients did not have a laboratory visit in their first short treatment course

**Fig 2:**
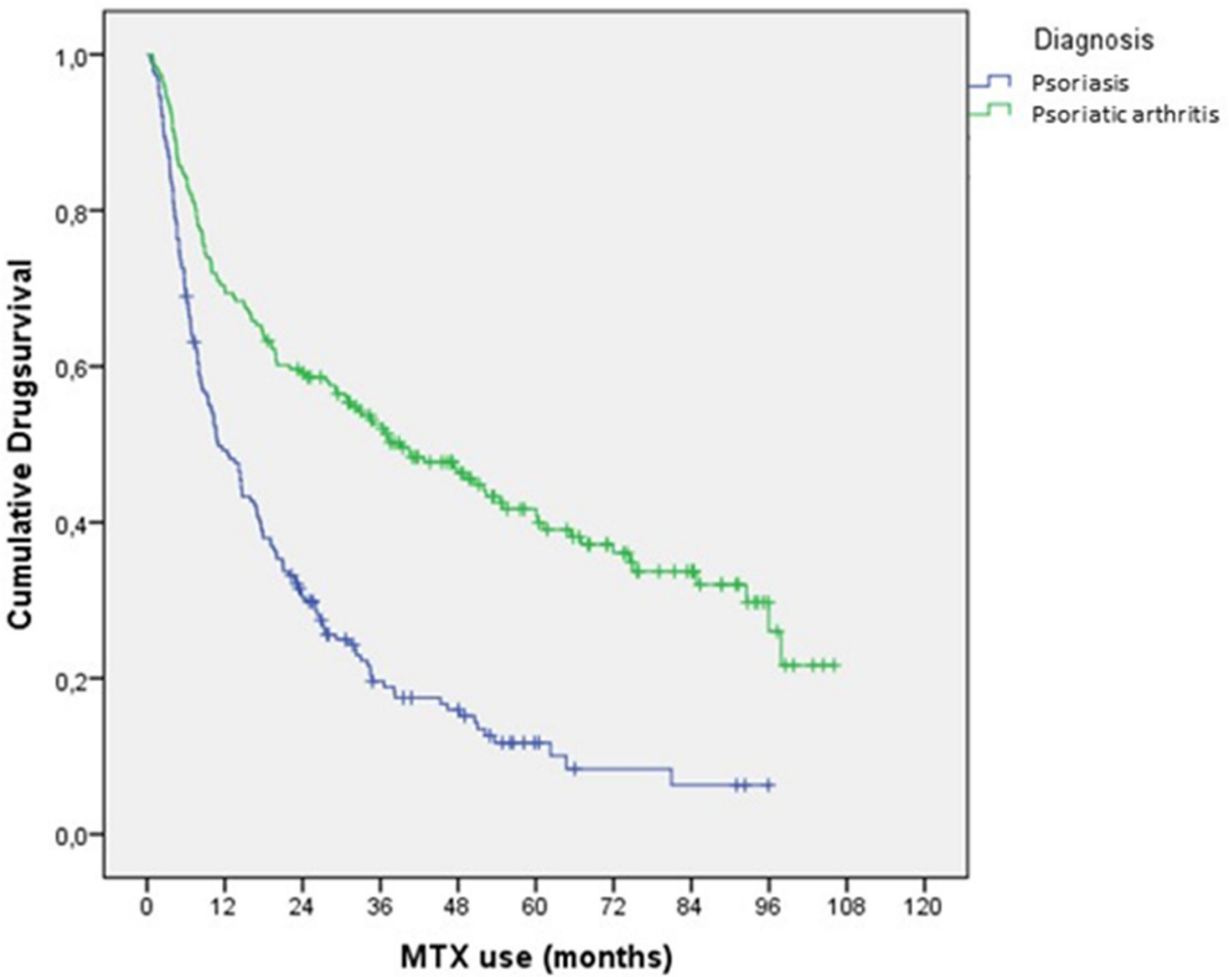
Continuation of methotrexate in first observed treatment course for psoriasis by dermatologists and psoriatic arthritis treated by rheumatologists (log-rank test: p<0.001).

Drug survival of the first treatment course is shown in Fig 2. In PsA patients it was 2.2-fold higher than in psoriasis patients (hazard ratio (HR) 2.2; 95% CI 1.7–2.8). A higher drug survival was found in those with prior treatment of MTX (HR 3.0; 95%CI 1.2–8.0) and also in those without prior treatment (HR 2.1; 95%CI 1.6–2.6). Rheumatologists commonly resumed MTX when it was temporarily withdrawn for toxicity, or when disease flared after a remission. Also, rheumatologists more often continued methotrexate when a biological disease modifying drug (b-DMARD) was started. This results in a highly prolonged overall use of methotrexate. In line with this observation psoriasis patients failing on methotrexate were more often switched to a b-DMARD: 86 out of 190 (45.3%) at an average of 23 months (range 3–80) after the first start of MTX, as opposed to 28 out of 196 (14.3%) PsA patients at 24 (2–83) months.

Reasons for withdrawal of MTX are summarized in Table 4. Four times more patients (15.8% vs 3.6%) with psoriasis compared to PsA were advised to stop MTX for abnormal laboratory results, almost exclusively elevated liver enzymes. In PsA, MTX was more often withdrawn for remission, and less frequently for ineffectiveness. Reduced leukocyte or thrombocyte counts were observed in 8 (4.2%) and 16 (8.2%) of patients with psoriasis and PsA respectively. These results induced dose adjustments of methotrexate or folic acid and led to withdrawal in a single patient, who appeared to have acute myeloid leukemia. Dermatologists also monitored leukocyte differentiation and serum albumin which did not result in values that induced stopping or dose adjustment of MTX.

**Table 4:** Reasons for withdrawal from the first course of MTX in patients with psoriasis or psoriatic arthritis.

	Psoriasis	Psoriatic arthritis
	N = 190	N = 196
Ineffectiveness of MTX	46 (24.2%)	31 (15.8%) *
Disease remission	15 (7.9%)	23 (11.7%)
Abnormal laboratory result	30 (15.8%)	7 (3.6%) **
Other drug toxicity, e.g. nausea	45 (23.7%)	41 (20.9%)
Death not related to MTX	2 (1.1%)	4 (2.0%)
End of follow-up, unrelated to disease	4 (2.1%)	1 (0.5%)
Comorbid conditions	4 (2.1%)	3 (1.5%)
Other	15 (7.9%)	10 (5.1%)
Continued MTX to censor date	29 (15.3%)	76 (38.8%)**

* P value < = 0.05;

** P-value <0.001

### Adverse events

SAE were observed over the first and later courses, regarding MTX treatment during 8105 person months in PsA, and 3865 person months in psoriasis patients. SAE leading to permanent withdrawal of MTX were observed in four PsA and three psoriasis patients on MTX treatment, resulting in an equal incidence rate (Table 5). In the psoriasis group two patients died from cardiovascular disease and one from gastric carcinoma. Cause of death in the PsA group was cancer in two patients, aortic aneurysm and sepsis in one each. In the psoriasis group, 8 patients had a hospital admission while using MTX, four of these for an infectious disease and therefore possibly related to MTX use. In the PsA group 14 patients had one or more hospital admissions, of which 6 were for infectious diseases. In 8 of the 10 patients hospitalized for infectious diseases, MTX treatment was resumed when the patient had recovered. In none of these SAEs abnormal laboratory monitoring results preceded the event.

**Table 5:** Serious adverse events (SAE) in patients while using methotrexate (first and later courses).

	Psoriasis	Psoriatic arthritis	P-value
	N = 190	N = 196	
SAE per treatment month, mean (SD)	0.0041 (0.024)	0.0038 (0.031)	0.9
Any SAE (%)	11 (5.8%)	18 (9.2%)	0.2
Death	3 (1.6%)	4 (2.0%)	0.7
Hospital admission	8 (4.2%)	14 (7.1%)	0.2

In three patients liver abnormalities contributed to the decision to stop MTX. In a patient with psoriasis without significant elevation of liver enzymes a liver biopsy after 4 years of treatment revealed non-specific changes, leading to a decision to end MTX treatment. In a patient with PsA a single elevated ALT led to the detection of liver adenoma, judged to be related to oral contraceptives. MTX was temporarily withdrawn and later resumed without further lab abnormalities. Another case with PsA had pre-existing liver enzyme abnormalities that appeared to be caused by liver cirrhosis after 8 weeks of treatment with MTX 10mg weekly. A causal relation with the drug was considered unlikely, but MTX was not restarted.

## Discussion

In this follow-up study of extensive monitoring of MTX treatment as advised in dermatological guidelines we observed earlier withdrawal for suspected liver toxicity compared to the monitoring policy in rheumatological guidelines. The most frequent reasons for withdrawal are ineffectiveness of MTX and abnormal laboratory results, both being much more present in psoriasis compared to PsA. Drug survival of MTX is considerably shorter when guided by dermatologists. The monitoring strategy applied by rheumatologists, with a follow-up reaching 8 years and almost 700 person years of observation, did not result in a higher number of serious adverse events caused by methotrexate. It is unlikely that the events observed in this study would have been prevented by more intensive laboratory tests. These observations provide a strong argument against the use of more frequent and multiple liver enzyme tests to monitor MTX.

Guidelines for monitoring MTX have a long history and are based on rare observations of serious adverse events. The advised strategies were not based on quantitative evidence. In a meta-analysis of 28 randomized controlled trials a twofold increased risk of elevated transaminases, but not of liver failure, cirrhosis or death was found. [13] This study was, however, limited to two years of MTX use.

The observed differences between psoriasis and PsA patients raise questions. First, one wonders if patients with psoriasis without arthritis are different from those with joint inflammations. From a pathophysiological point of view the opposite can be expected, since more systems are affected, and patients with arthritis are more prone to use additional medication. In our hospital a diagnosis of PsA without clinical psoriasis is rarely made. Therefore the argument that psoriasis patients are more prone to liver abnormalities is not valid. Unfortunately we were unable to obtain reliable data regarding body mass index (BMI), which may affect liver enzymes. However, it is unlikely that psoriasis patients with arthritis have lower BMI than patients without musculoskeletal symptoms. Comparisons with rheumatoid arthritis did not reveal a specific risk of adverse events associated with psoriasis. [19,20] As illustrated in our study, the rate of elevations of ALT, which was tested in psoriasis and PsA, was equal in the first course of MTX observed. The protocols of rheumatologists and dermatologists diverge mainly in the use of one vs three liver function tests, and the cut-off levels to withdraw MTX: 2 vs 3 times the upper limit of normal. As illustrated in Table 3, the use of additional liver function tests resulted in more abnormalities leading to drug withdrawal. Detailed analysis of ALAT exceeding 2 or 3 times the upper limit of normal did not reveal significant differences between PsA and psoriasis patients.

Second, it should be noted that dermatologists used slightly lower doses of both methotrexate and folic acid. The folic acid dose of 5 mg used by dermatologists was proven to be effective in the trial by van Ede et al [21]. It is not known if higher doses result in further reduction of liver enzyme abnormalities. These differences are small, however, and we do not expect a significant effect on liver toxicity. The low doses of MTX that were used by dermatologists may be an explanation for the high percentage (24%) that stopped MTX for ineffectiveness. Differences in dosing of MTX between dermatologists and rheumatologists are not unique, and were also observed in a Canadian survey. [22]

Given the lack of a physiological difference, the different monitoring guideline is a prominent explanation for the observed difference of 16% vs 4% in abnormal laboratory results leading to withdrawal of MTX. Noticing details in the records, rheumatologists handled laboratory abnormalities often by a repeat test (which often was normal), increasing the dose of folic acid, or reducing the dose of MTX. Dermatologists responded more often by immediately switching to another drug. Thus practice variation, and not differences between patients, appeared to be a major driver of drug survival.

The strength of this unique study is the long observation period in a large number of patients, which allows comparison of the safety of the monitoring policies of rheumatologists and dermatologists. The frequency of abnormal test results in our study population is in line with a recent meta-analysis. [23] The setting of a large regional hospital, with electronic records comprising data from all specialties, ensured complete follow-up of patients. This scrutiny did not reveal serious adverse events that could have been prevented by more intensive monitoring, which supports the long-term safety of the rheumatological guideline.

A limitation of our study is that the numbers do not allow firm conclusions on adverse events with an incidence less than 1%. In RA, a 5 year risk of serious liver disease has been estimated below 1/1000, and this may even be an overestimation. [13,24] Evidence for the effectiveness of monitoring strategies to prevent long-term liver damage therefore requires thousands of patients. Cost-effectiveness of the elaborate monitoring advised in dermatological guidelines is unlikely. Our observations underline, that apart from the obvious costs of more tests, abnormal test results lead to early withdrawal of MTX. Moreover, in patients with psoriasis compared to PsA, MTX was three times more often replaced by a b-DMARD, which further increases the cost for patients and society.

In conclusion, our data illustrate that extensive monitoring of MTX, as advised in dermatological guidelines for psoriasis, results in more abnormal test results and early withdrawal. It is unlikely that patients with psoriasis require more intensive screening than patients with PsA or RA. The safety of MTX is comparable and a large body of evidence in RA indicates that the risk of serious liver damage is very low. However, further research to evaluate the safety of less intensive MTX monitoring is desirable. Reconsideration of dermatological monitoring guidelines for MTX may enhance its effective use in psoriasis and reduce costs.
